# Discordance Between Human Papillomavirus Twitter Images and Disparities in Human Papillomavirus Risk and Disease in the United States: Mixed-Methods Analysis

**DOI:** 10.2196/10244

**Published:** 2018-09-14

**Authors:** Yuki Lama, Tao Chen, Mark Dredze, Amelia Jamison, Sandra Crouse Quinn, David A Broniatowski

**Affiliations:** 1 Department of Family Science School of Public Health University of Maryland, College Park College Park, MD United States; 2 Center for Language and Speech Processing Johns Hopkins University Baltimore, MD United States; 3 Center for Health Equity School of Public Health University of Maryland, College Park College Park, MD United States; 4 Department of Engineering Management and Systems Engineering The George Washington University Washington DC, DC United States

**Keywords:** disparities, health communication, HPV vaccines, image tweet, public health communication, social media, Twitter, visual communication

## Abstract

**Background:**

Racial and ethnic minorities are disproportionately affected by human papillomavirus (HPV)-related cancer, many of which could have been prevented with vaccination. Yet, the initiation and completion rates of HPV vaccination remain low among these populations. Given the importance of social media platforms for health communication, we examined US-based HPV images on Twitter. We explored inconsistencies between the demographics represented in HPV images and the populations that experience the greatest burden of HPV-related disease.

**Objective:**

The objective of our study was to observe whether HPV images on Twitter reflect the actual burden of disease by select demographics and determine to what extent Twitter accounts utilized images that reflect the burden of disease in their health communication messages.

**Methods:**

We identified 456 image tweets about HPV that contained faces posted by US users between November 11, 2014 and August 8, 2016. We identified images containing at least one human face and utilized Face++ software to automatically extract the gender, age, and race of each face. We manually annotated the source accounts of these tweets into 3 types as follows: government (38/298, 12.8%), organizations (161/298, 54.0%), and individual (99/298, 33.2%) and topics (news, health, and other) to examine how images varied by message source.

**Results:**

Findings reflected the racial demographics of the US population but not the disease burden (795/1219, 65.22% white faces; 140/1219, 11.48% black faces; 71/1219, 5.82% Asian faces; and 213/1219, 17.47% racially ambiguous faces). Gender disparities were evident in the image faces; 71.70% (874/1219) represented female faces, whereas only 27.89% (340/1219) represented male faces. Among the 11-26 years age group recommended to receive HPV vaccine, HPV images contained more female-only faces (214/616, 34.3%) than males (37/616, 6.0%); the remainder of images included both male and female faces (365/616, 59.3%). Gender and racial disparities were present across different image sources. Faces from government sources were more likely to depict females (n=44) compared with males (n=16). Of male faces, 80% (12/15) of youth and 100% (1/1) of adults were white. News organization sources depicted high proportions of white faces (28/38, 97% of female youth and 12/12, 100% of adult males). Face++ identified fewer faces compared with manual annotation because of limitations with detecting multiple, small, or blurry faces. Nonetheless, Face++ achieved a high degree of accuracy with respect to gender, race, and age compared with manual annotation.

**Conclusions:**

This study reveals critical differences between the demographics reflected in HPV images and the actual burden of disease. Racial minorities are less likely to appear in HPV images despite higher rates of HPV incidence. Health communication efforts need to represent populations at risk better if we seek to reduce disparities in HPV infection.

## Introduction

Approximately 23,300 women and 16,500 men develop human papillomavirus (HPV)-related cancer annually in the United States, many of which could have been prevented with the HPV vaccination [1]. The US Centers for Disease Control and Prevention (CDC) recommends routine vaccination, with a series of 3 doses of HPV vaccine, for preteen girls and boys starting at the age of 11 years with catch-up vaccination for 18-26-year-olds who have not been previously vaccinated [2].

National rates of the HPV vaccination remain suboptimal with only 60.4% of adolescent girls (aged 13-17 years) initiating and 49.5% completing the series and 56.0% of adolescent boys initiating and 43.4% completing—well below Healthy People 2020 objective to increase HPV 3-doses vaccination series completion for adolescents aged 13-15 years to 80% by 2020. CDC findings indicate that these rates are comparably low for childhood vaccinations, for example, tetanus, diphtheria, and pertussis (88.0%), measles, mumps, and rubella (measles, mumps, and rubella; 90.9%), and hepatitis B (91.4%) [1]. Furthermore, disparities exist by race, ethnicity, and gender. Uptake rates have been low for African American individuals in comparison with non-Hispanic white individuals [3-6]. Black women were less likely to finish the series compared with their white counterparts [4,7-11]. Gelman et al found lower rates of uptake among African American girls (18.2%) compared with non-Hispanic white girls (33.1%). This issue is compounded because the risks of nonvaccination are higher for people of color [3]. Minority women are more likely to die from cervical cancer with the highest incidence rate of cervical cancer (13.2 per 100,000 women) of Hispanic origin, followed by African American women (9.8 per 100,000 women) [12,13]. According to findings of Mourad et al, HPV-related cancer rates have been increasing in men and currently exceed cervical cancer rates, for example, oropharyngeal cancer among men (7.8 per 100 000) compared with cervical cancer in women (7.4 per 100 000) [14]. Black men have the highest rates of HPV-associated anal cancer, and Hispanic men have higher rates of penile cancer than non-Hispanic men [13,15]. In addition, vaccine coverage remains troublingly low for males of all races and ethnicities [1,3]. Because men are increasingly affected by anal and oropharyngeal (head and neck) cancers, suboptimal HPV vaccination is a lost opportunity for cancer prevention. Given the serious consequences of low vaccination rates, health communication efforts should focus on high-risk populations.

Targeted and tailored communication methods can be utilized to enhance the HPV vaccination uptake for high-risk groups [16]. Targeted approaches customize messaging toward subgroups based on shared characteristics (eg, race and gender), allowing the distribution of messages in strategically and cost effectively. Tailored approaches focus on fitting the message to meet the needs of an individual to effectively influence health behaviors. Literature indicates that targeted and tailored messages increased perceived cancer risk and cancer information compared with generic messages [17,18]. It is clear that including representative images of at-risk groups in health communication images increases awareness, relevance, and impact of a health issue on the group members. Indeed, messages reflecting the images of the intended audience are critical to promote the HPV vaccination uptake and reduce observed disparities.

Social media can be utilized for health promotion for minorities. Twitter has been used extensively to study vaccine narratives, including those related to HPV, measles, and influenza [19-22]. Given Twitter’s user base of over 500 million and publicly available posts on HPV, it is a strategic site for health communicators to track HPV sentiment and target HPV vaccine messaging [23-25]. Massey et al examined tweet sentiment and content for 193,379 tweets from August 1, 2014 to July 31, 2015; positive tweets were more likely to mention prevention, whereas negative tweets increased the focus on side effects [20]. Dunn et al observed 258,418 tweets from October 2013 to October 2015 to measure information exposure differences and corresponding HPV vaccine coverage differences across states; results indicated that the lower HPV vaccine coverage correlated with the negative HPV sentiment from mainstream news, highlighting the influence of the media on the HPV vaccine uptake [26]. In addition, almost one-quarter of internet users, many of whom are from racial and ethnic minorities [27], use Twitter. Thus, Twitter images are an opportune and underutilized resource for studying health communications related to the HPV vaccination.

Given the importance of social media platforms for health communication, we examined HPV vaccine messages. Specifically, we focused on image tweets, which tend to receive more shares than nonimage tweets [28,29]. Previous health communication research has shown the power of imagery [30-32]. We extended this work by examining the demographics of the individuals pictured in HPV Twitter images. In particular, we used facial recognition technology, a product of recent advances in the computer vision subdiscipline of computer science, which builds high-quality image analysis algorithms [33-39]. These methods are accessible to public health researchers through companies who provide cost-effective image analysis services. However, to the best of our knowledge, no studies have explored HPV-related disparities using Twitter images and facial recognition technology. We, therefore, showed how these methods can enable large-scale health-related image analyses. Precisely, we evaluated Twitter images to investigate to what extent Web-based health communication represents minority groups that are disproportionately affected by HPV-associated morbidities.

This study’s implications have the potential to inform the development of more culturally relevant messaging, aligning health promotion imagery salient to intended audiences of those disproportionately affected by HPV-related negative health outcomes. Public health agencies can utilize findings to improve health communication approaches on social media to reduce disparities of HPV-associated disease for all racial and ethnic groups.

Hence, this study aims to observe whether the demographics reflected in HPV images on Twitter reflect the populations suffering from the actual burden of disease by the gender and race and determine to what extent individual users, governmental users, and organizational accounts utilized images that reflect the populations bearing the burden of disease in their health communication messages.

## Methods

### Dataset

**Figure 1:**
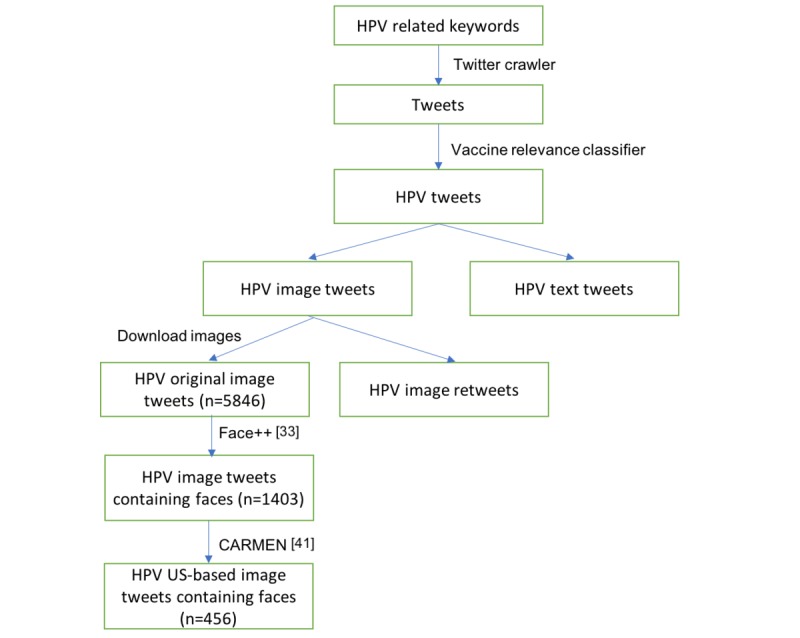
The flowchart of our human papillomavirus (HPV) image tweet data collection.

We constructed a corpus of tweets relevant to HPV following the approach described in a study by Chen et al [29]. Figure 1 shows the flowchart of this data collection process. We first collected tweets that contained any of the 2 terms related to HPV (namely, “HPV” and “papillomavirus”) using the Twitter streaming API from November 11, 2014 to August 8, 2016, and further filtered out vaccine-irrelevant tweets using a statistical classifier [40]. This support vector machine classifier aims to distinguish vaccine-relevant tweets from vaccine-irrelevant tweets. It was trained on 1899 manually annotated tweets and achieved good performance (precision=0.96; recall=0.91; *F*_1_=0.93) [40]. The time frame reflects image tweets collected from a previous vaccine images tweet analysis [29]. We then downloaded images for original image tweets, excluding retweets that had duplicate images.

We next used Face*++* [33], a Web-based face recognition tool, to automatically categorize images containing faces and facial properties (eg, gender, age, and race). We selected Face++ because it has a high reported accuracy in the literature and supports race identification. We found that 25.8% of images had at least one face.

In addition, we obtained the locations of these image tweets using the CARMEN geolocation tool [41]. CARMEN infers the location of a tweet from the user’s profile and geotags in the metadata of the tweet. CARMEN has previously resolved location for 44.45% of tweets and correctly labeled the tweet location to within 250 miles of its true origin 75.27% of the time [41]. For our HPV image tweets, 32.5% of tweets were from the United States, and the location of 48.7% tweets was unknown. Our final dataset contained 456 HPV US-based image tweets containing at least one face.

### Face Attributes Identification

We obtained 3 face attributes, gender (female or male), age (an integer), and perceived race (white, black, Asian, or ambiguous, which are the only 4 categories provided by Face++), of HPV images from Face++. Face++ has been widely used for face detection, recognition, and face attribute identification in social images like Twitter [35-38] and on major search engine images [34]. As reported previously, Face++ achieved a true positive rate of 85% in face detection with a false positive rate of 0.1 [39], 88% accuracy in gender recognition, and 79% accuracy in the race recognition [42].

Exact age estimation is a difficult task for both machines and humans. Face++ has a mean absolute error of 11.0 for exact age estimation [43] but a much higher accuracy (>93%) when it groups ages into categories (<18 years, 18-35 years, and >35 years) [44]. To allow the estimated age to be more robust, we organized age into 5 groups, namely, infant (0-2 years), child (3-8 years), youth (9-26 years, the recommended age to take the HPV vaccination by CDC), adult (27-64 years), and senior (>64 years).

To further validate the performance of Face++, we manually annotated the perceived gender, race, and age group attributes of all visible faces in each HPV image. The 2 annotators first worked independently and then resolved discrepancies by consensus. This process also helped us to gain insights from the HPV images as well as challenges of classifying the race, which is further detailed in the Discussion section.

### Analysis

We performed a mixed-methods analysis for the demographics reflected in the 3 face attributes. Based on the gender of faces, we first categorized images into 3 groups as follows: images that only have female faces, those that only have male faces, and those that have faces of both genders. We then compared the race and age distribution across these 3 groups. Categories chosen for the race were consistent with the current CDC standards for data collection and included Asian or Pacific Islander, black, and white individuals [45]. Owing to the technical limitations of Face++ of identifying the race and ethnicity, our racial classification system was streamlined (by dropping the racial classification for American Indian and Alaskan Native and the question of Hispanic ethnicity [46]) and embraced ambiguity (by adding a category for “ambiguous”). We acknowledge that the race and gender are nuanced social constructs that are particularly challenging to classify (see Discussion section). In this study, the race and gender, as perceived by researchers, were used to highlight differences in Twitter images. Racial perception is an appropriate measure as we seek to understand how health communications will be perceived. Notably, the complexities of reifying the race and gender are beyond the scope of this study.

Our analysis focused on images containing youths (recommended HPV vaccine recipients) or adults (eg, parents or health care providers). In addition, we examined the association between facial attributes and the source of the image tweet; for example, do government users or users with a health focus tend to post faces that reflect the actual burden of HPV diseases? To answer such questions, we manually examined (first annotated by one author of this paper, and then checked by 2 additional authors) all the source profiles of our HPV dataset (298 unique sources in total). We categorized these sources into 3 types, namely, government (eg, CDC and local health departments), nongovernment organizations (including health-related organizations), and individuals. We further stratified sources into health-related (eg, health care provider) or news-related categories. We then compared facial attributes across the resulting source categories.

## Results

### Face++ Versus Manual Annotation

Manual annotation identified more faces (1219) than Face++ (999). The discrepancy is primarily caused by images with multiple small, blurry, or nonfront-facing faces. Such faces are still distinguishable by humans but are rather difficult for automatic tools like Face++ to recognize. Because the subject of an image is usually the clearest and largest face, we believe Face++ has detected most important faces in our dataset (see Multimedia Appendix 1).

**Figure 2:**
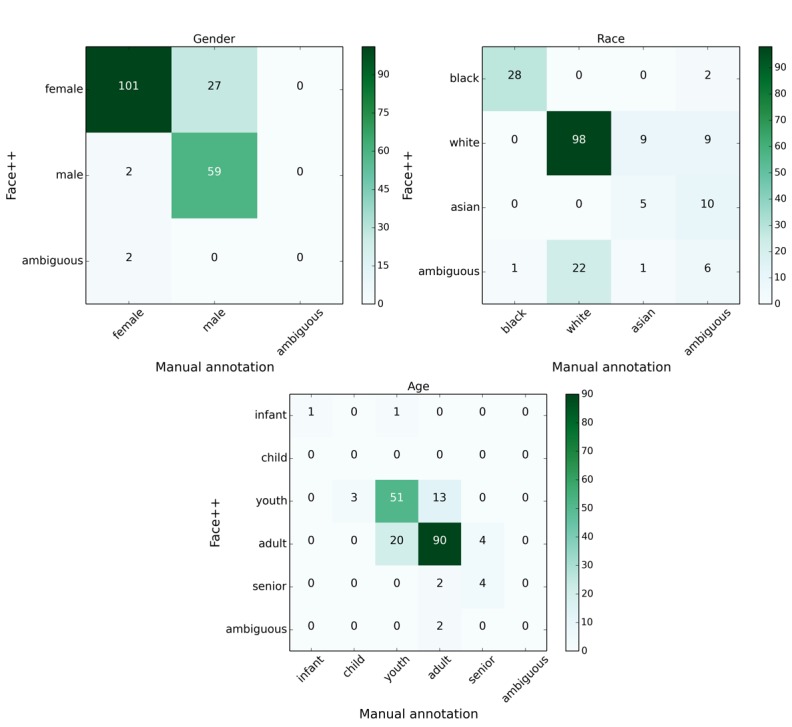
The confusion matrices showing the performance of Face++ against manual annotation.

Table 1 details the distribution of 456 HPV images by the face count. Most images—55.6% according to manual annotation—contained multiple faces. Owing to the lack of face positions in manual annotation, it is difficult to align the annotated faces by human annotators and Face++ when multiple faces are presented in an image. Therefore, we limited the direct comparison of 2 annotations to images with a single face. Face++ achieved accuracy values of 84.7%, 76.4%, and 85.1% with respect to gender, race, and age, respectively, when compared with manual annotation, ignoring images with ambiguous labels. To gain an in-depth understanding of the performance of Face++, the confusion matrix is presented in Figure 2.

**Table 1:** The number and distribution of images by the face count.

Number of faces in the image	Face++, n (%)	Manual annotation, n (%)
1	279 (60.5)	196 (44.4)
2	72 (15.6)	99 (22.4)
3	43 (9.3)	58 (13.2)
4	22 (4.8)	18 (4.1)
>4	45 (9.8)	70 (15.9)

For gender, Face++ mistakenly labeled 27 female faces as male when considering manual annotation as ground truth. Of these 27, 12 (44.4%) were actually black women. For the race, Face++ identified 100% of black faces but had difficulty in differentiating white and Asian faces. Our human annotators acknowledged the same difficulty, leading them to label the race of 27 faces as ambiguous. Regarding age, we identified youth and adult age categories to have the greatest discrepancy between Face++ and manual annotation. From manual annotation, we acknowledge the challenge in distinguishing between age cutoffs (eg, 26 vs 27 years old). As such, the actual performance of Face++ on age has an accuracy of >85.1%. Overall, Face++ is reliable in detecting important faces and recognizing the facial properties (age, gender, and race) for our HPV images.

### Gender, Age, and Race

Considering manual annotation is more accurate than Face++, we only detail the results of manual annotation in the following tables. Our results from manual annotation and adoption of Face++ were broadly consistent, highlighting the potential for the automated face analysis utilization in public health research (see Multimedia Appendix 1). We first examined the gender of faces. At the broader image level in our dataset, 53.6% of images had only female faces, 17% had only male faces, 27.9% had both genders, and the remaining 1.6% had ambiguous gender or did not have a face (we excluded these 1.6% images in the following analysis). At the individual face level in our dataset (Table 2), 71.70% (874/1219) of faces were female, 27.89% (340/1219) were male, and the rest 0.41% (5/1219) had ambiguous gender (eg, infants’ faces).

Tables 3 and 4 show the number (percentage) of faces by the age and race, respectively. Overall, youth (616/1219, 50.53%) and adults (578/1219, 47.41%) were the 2 primary age groups, but the detailed distributions varied in each gender group. For instance, within the youth category, the majority of images included faces of both genders (365/616, 59.3%) and female faces (214/616, 34.3%), whereas in the adult category, the largest portion was female images (285/578, 49.3%), followed by both genders (219/578, 37.9%).

Looking at the race (Table 4), the majority of faces (795/1219, 65.22%) were white, followed by black (140/1219, 11.48%) and Asian (71/1219, 5.82%), whereas 17.47% (213/1219) of faces were racially ambiguous. For the overall gender distribution, female faces were predominantly represented across races (331/795, 42.39% white; 71/140, 50.71% black; and 34/71, 47.89% Asian) compared with male faces (7/140, 5.0% black; 75/795, 9.43% white; and 8/71, 11.27% Asian). In addition, we observed that over half (121/213, 56.80%) of faces with ambiguous races appeared in images with both genders.

**Table 2:** The number and percentage of demographics at face level by manual annotation.

Face characteristics		n (%)
**Gender**		
	Female only	874 (71.70)
	Male only	340 (27.89)
	Ambiguous	5 (0.41)
**Age group**		
	Infant	0 (0.00)
	Child	10 (0.82)
	Youth	616 (50.53)
	Adult	578 (47.41)
	Senior	13 (1.07)
	Ambiguous	2 (0.16)
**Race**		
	Black	140 (11.48)
	White	795 (65.22)
	Asian	71 (5.82)
	Ambiguous	213 (17.47)
Total faces		1219 (100)

**Table 3:** The number and percentage of faces in each age group by manual annotation.

Age group	Faces in female-only images, n (%)	Faces in male-only images, n (%)	Faces in images with both genders, n (%)	Total faces, n
Infant	0 (0.0)	0 (0.0)	0 (0.0)	0
Child	4 (40.0)	1 (10.0)	5 (50.0)	10
Youth	214 (34.3)	37 (6.0)	365 (59.3)	616
Adult	285 (49.3)	74 (12.8)	219 (37.9)	578
Senior	6 (46.2)	1 (7.7)	6 (46.2)	13
Ambiguous	2 (100)	0 (0.0)	0 (0.0)	2
Total	511 (100)	113 (100)	595 (100)	1219

**Table 4:** The number and percentage of faces in each race by manual annotation.

Race	Faces in female-only images, n (%)	Faces in male-only images, n (%)	Face in images with both genders, n (%)	Total faces, n (%)
Black	71 (50.7)	7 (5.0)	62 (44.3)	140 (11.48)
White	337 (42.4)	75 (9.4)	383 (48.2)	795 (65.22)
Asian	34 (47.9)	8 (11.3)	29 (40.8)	71 (5.82)
Ambiguous	69 (32.4)	23 (10.8)	121 (56.8)	213 (17.47)
Total	511 (100)	113 (100)	595 (100)	1219 (100)

**Table 5:** The demographic distribution for youth and adult by manual annotation.

Gender and age			Black, n (%)	White, n (%)		Asian, n (%)		Ambiguous, n (%)
**Female**								
	Youth	28 (13.1)		145 (67.8)	10 (4.7)		31 (14.5)	
	Adult	43 (15.1)		182 (63.9)	24 (8.4)		36 (12.6)	
**Male**								
	Youth	6 (16.2)		24 (64.9)	0 (0.0)		7 (18.9)	
	Adult	1 (1.4)		49 (66.2)	8 (10.8)		16 (21.6)	
**Both genders**								
	Youth	49 (13.4)		203 (55.6)	19 (5.2)		94 (25.8)	
	Adult	11 (5.0)		171 (78.1)	10 (4.6)		27 (12.3)	

Finally, we focused on youth and adult age faces and examined the gender and racial breakdown for the 2 age groups (detailed in Table 5). In addition to the consistently higher proportion of white faces across all gender and age categories, we observed a higher percentage (171/219, 78.1%) of white adult faces. In images with black faces, a black adult was rare in images with only male faces (1/74, 1.4%) or those with both genders (11/219, 5.0%). For Asians, we did not observe any Asian youth in images only with male faces.

### Source of Images

**Figure 3:**
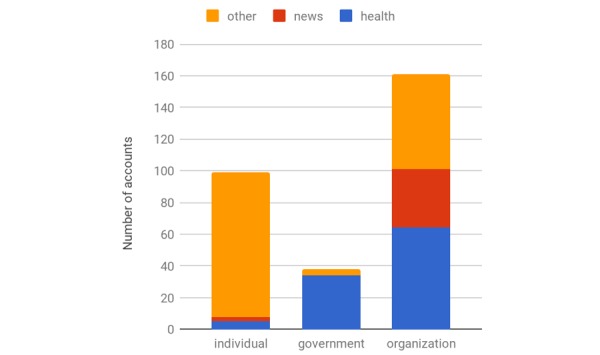
Distribution of sources.

Figure 3 shows the source categorization results. The authors manually annotated sources into 3 broad categories (ie, organizations, government, and individual). Source categories were further annotated by 3 specific topics (ie, news, health, and other). The majority (161/298, 54.0%) of sources were organizations, followed by individuals (99/298, 33.2%) and government (38/298, 12.8%). Regarding the topic, 13.4% (40/298) of sources were news related (eg, Huffington Post Blog and Business Insider) and 34.6% (103/298) were health related. We found that government sources were primarily health related (34/38, 90%) and organizations were more health related (64/161, 39.8%) than news related (37/161, 23.0%), whereas individual sources did not have a strong health or news focus (91/99, 92% of users had other topics).

Multimedia Appendix 2 shows the breakdown of demographic results by the source. Organization sources used more images with white faces. For instance, 96.6% (28/38) of female youth and 100% (12/12) of adult male images posted by news organization sources were white. These findings were significant, especially given the influence of media in promoting vaccination to reduce disparities (see Discussion).

Governmental health sources primarily posted images that had both genders, whereas news organization sources preferred female faces. Health-related organizations utilize many youth faces in images with both genders, and individual sources had an equal likelihood of posting female or mixed gender images. For the race, governmental health agencies tended to post a large proportion of black female faces in both youth (6/24, 25%) and adult age groups (4/20, 20%), more frequently than other sources (Table 5). Furthermore, governmental health sources posted a high proportion of youth faces of ambiguous race (5/24, 21% female; 3/15, 20% male; and 28/92, 30% both genders).

## Discussion

### Principal Findings

This study examined the content of HPV Twitter images. Despite reflecting demographics similar to US Census data, our results show a distinct difference between the demographics reflected in Twitter images and the actual burden of disease by the race and gender. Additional Census comparison figures are included in the Multimedia Appendix 1.

First, our analyses demonstrate that male faces are significantly underrepresented in HPV images (9.3%), particularly for youth, the recommended group for the HPV vaccination—34.3% girls versus 6.0% boys (Table 3); this is consistent with the observed lower uptake of the HPV vaccine among males. Currently, 37.5% of male youth were up to date with recommended HPV vaccination series compared with 49.5% for female youth [1], highlighting the need for further targeted HPV vaccine promotion toward male youth. Presenting the HPV vaccination for males as cancer and genital wart prevention has helped increased acceptance among males [47]. Continued efforts of increasing the awareness and representation of both genders in HPV communication are crucial in preventing HPV-associated cancer that affect men and women alike. Second, Twitter images had significantly fewer images of African American individuals even though African American individuals are more likely to be affected by HPV-associated cancer. Consistent with US Census data, our results demonstrate that white faces represented 65.22% (795/1219) of the images compared with 11.48% (140/1219) African American faces and 5.82% (71/1219) Asian faces [48]. However, of critical importance is that African American individuals are disproportionately affected by HPV-related diseases and are less likely to complete the HPV vaccination series compared with their white counterparts [4]. Furthermore, black adults are more likely to use Twitter than other racial groups, making Twitter a pertinent health communication platform to target parents of black youth [27]. Our findings suggest that despite known disparities, health communication efforts on Twitter have not sought to include the representation of African American individuals in targeted communications that could address racial disparities in the HPV vaccination [27].

Of note, images that included both females and males were usually represented by groups of youth (365/616, 59.3%), whereas for female-only and male-only faces, images were typically youth accompanied by adults who were more likely to be male (65.5%). From manual annotation, annotators noted that adults were typically represented by parents or health care providers. From the limited number of male faces in the sample (340/1219, 27.9%), male adults were twice as likely to be represented (74/578, 12.8%) compared with male youth (37/616, 6.0%), highlighting the insufficient communication efforts to target male youth for the HPV vaccination. Third, our results showed that the frequency of Twitter images posted from individual, organization, and government sources also reflected discrepancies in the minority representation. Individual sources shared more images with females (n=117) than males (n=37), suggesting the majority of users perceive HPV as an exclusively female issue. Organization users (health- or news-related) depict almost exclusively white faces.

The influence of Web-based news organizations is significant, especially given that 62% of American individuals access the internet for health information [49]. Although it is not their role to promote health behaviors, Web-based news sources are certainly influential in reporting and detecting emerging trends and outbreaks [22,23]. By depicting mostly white faces in Web-based HPV communication, these organizations might be reinforcing an inaccurate message about who is most at risk and failing to address groups that would also benefit from vaccination.

These findings are distinct from government images, which could be attributed to the differing incentives and roles of the media related to promoting vaccination compared with the government. In addition to having a lower presence tweeting black HPV images, government sources, which were typically local, state, and federal public health agencies, also had an uneven distribution of gender and racial representation within aforementioned images. Males represented in the images were overwhelmingly white—80% (12/15) of youth and 100% (1/1) of adults. Government HPV images with both genders included predominantly racially ambiguous youth (5/24, 21% female; 3/15, 20% male; and 28/92, 30% both gender), which may reflect an intentional choice to reach a more diverse audience, especially given the increasing number of multiracial youth in the Census [48]. However, the greater representation of racially ambiguous youth inadvertently fails to address racial and ethnic disparities in HPV health outcomes by underrepresenting those with the highest risk; this is especially critical given the role of governmental public health agencies in disseminating relevant health information.

In health communication, there is frequently an emphasis on the power of culturally sensitive communication, defined by Betsch et al as “the deliberate and evidence-informed adaptation of health communication to the recipients’ cultural background to increase knowledge and improve preparation for medical decision making and to enhance the persuasiveness of messages in health promotion” [50,51]. The aim is to create greater congruency between the health promotion messages and the recipient’s existing cultural context with an overall goal of increasing the effectiveness of messaging [52]; this can be accomplished through either targeting (aimed at the general cultural group) or tailoring (aimed at specific individuals within a cultural group). Visual representation of racial and ethnic minorities would represent a minimal effort at targeting high-risk groups and although it is not sufficient to guarantee the vaccine uptake, the exclusion of racial and ethnic minorities and males from images used in communication about the HPV vaccination is likely to perpetuate disparities in uptake and disease.

Our findings demonstrate discordance between HPV images on Twitter and those at the greatest risk of HPV-associated cancer. Twitter can be harnessed to disseminate HPV messages aimed at racial minorities who are more likely to be Twitter users [27]. Public health agencies would benefit from formative research with minority youth and their parents to improve Web-based health communication strategies to reduce the burden of HPV-associated cancer for all racial and ethnic groups.

### Limitations

There are limitations to this observational study. In this study, we also collected data for specific time points that yielded a relatively small sample of Twitter images, which speaks to the generalizability of findings. However, we found that characterizing the race, ethnicity, and age of faces was particularly challenging with both automated image analysis algorithms and manual annotation. Nearly half of the errors produced by Face++ were related to the misclassification of black female faces. Consistent with these results, Buolamwini and Gebru evaluated commercial gender classification algorithms and found that darker females have highest misclassification error rates (20.8%-34.7%) than darker males (0.7%-12.0%), lighter females (1.7%-7.1%), and lighter males (0.0%-0.8%) [53]. These findings highlight the limitations of automated image analysis for women of color, which have marked implications for real-world applications of this technology.

Self-identification is considered the gold standard for all race and ethnicity variables. In this instance, self-identification was not possible, and the race was ascribed by researchers engaged in manual annotation and through algorithms designed for this purpose. Unfortunately, the automated image analysis and, to a lesser extent, the manual annotation are dependent on the stereotypical phenotypic expressions of racialized features. The increasing population of mixed-race individuals, the complexity of the race and ethnicity with Latino populations, and indeed, the recognition that the race is only a social construct make an accurate determination of racial categories difficult. Furthermore, similar constraints made it difficult to consider any nonbinary expressions of gender. Thus, the potential for error of categorizing faces by specific age and race and gender needs to be considered in this study and future research.

### Conclusions

This study provides insights into racial and gender differences in HPV images on Twitter. Findings can inform imagery-driven health communication strategies to increase the vaccine uptake to mitigate negative health outcomes, particularly within the context of social media. Culturally sensitive communication, which would include increased representation of minorities in images, may enhance the salience of HPV messaging to populations disproportionately affected by HPV-related health outcomes.

## Appendix

Multimedia Appendix 1 The supplementary figures and tables for Census statistics and Face++ results.

Multimedia Appendix 2 The gender, age, and race distribution in different source group by manual annotation.

## Abbreviations

CDC: Centers for Disease Control and Prevention
HPV: human papillomavirus
