# Supplementary material for: Discordance Between Human Papillomavirus Twitter Images and Disparities in Human Papillomavirus Risk and Disease in the United States: Mixed-Methods Analysis
Source: J Med Internet Res. 2018 Sep 14;20(9):e10244. doi: 10.2196/10244 (PMC6231890; doi:10.2196/10244)
Supplement: Multimedia Appendix 1 [file jmir_v20i9e10244_app1.pdf]

Discordance between HPV Twitter Images and Disparities in HPV Risk and Disease: Mixed Methods Analysis

Multimedia Appendix 1

Census Comparisons

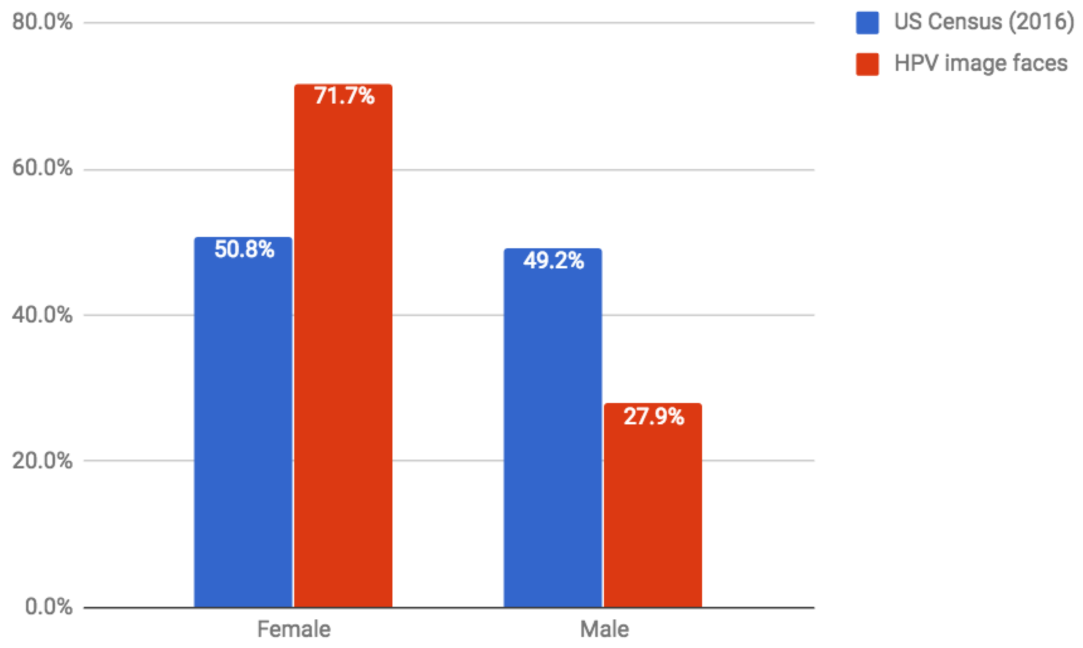

Figure 1: The gender distribution in US Census (2016) [1] and the faces of HPV images.

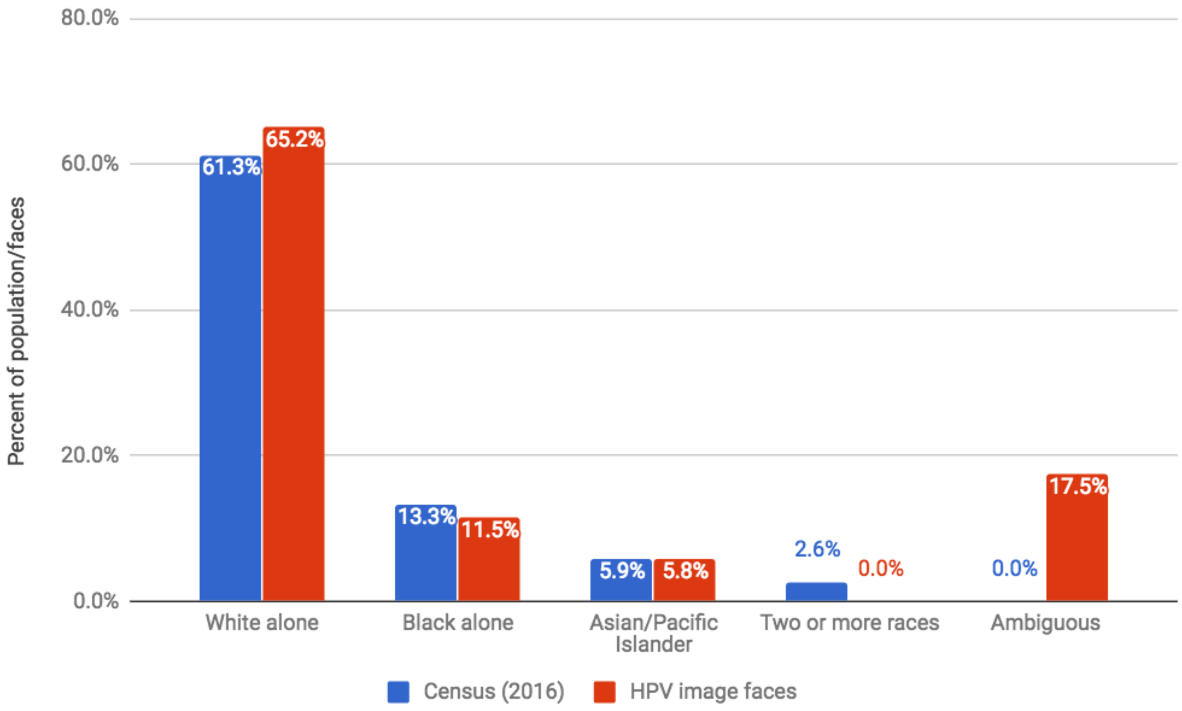

Figure 2: Race Origin in United States (Census, 2016) [2] vs. Race in HPV image faces.

Table 1: Percentages and Rate of HPV-Associated Cancers by Sex and Race (United States, 2009–2013) [3].

**Race**	**Census estimates (2016)**	**HPV image faces**	**Overall HPV-associated cancer incidence rate (per 100,000 person)**	**Female HPV-associated cancer incidence rate (per 100,000 person)**	**Male HPV-associated cancers incidence rate (per 100,000 person)**
While alone	61.3%	65.2%	12.0	13.7	10.3
Black alone	13.3%	11.5%	12.1	14.3	9.4
Asian/Pacific Islander	5.9%	5.8%	5.3	7.6	2.7
Two or more races	2.6%	0.0%	N/A	N/A	N/A

Face++ Results

Table 2: The number and distribution of faces in each age group by Face++.

**Age Group**	**Faces in female only images**	**Faces in male only images**	**Faces in images with both genders**	**Total faces**
Infant	2 (50.0%)	0 (0.0%)	2 (50.0%)	4
Child	12 (20.0%)	4 (6.8%)	43 (72.3%)	59
Youth	236 (44.6%)	51 (9.6%)	242 (45.7%)	529
Adult	185 (46.7%)	96 (24.4%)	113 (28.7%)	394
Senior	6 (46.2%)	4 (30.8%)	3 (23.1%)	13
**Total**	511	113	595	999

Table 3: The number and percentage of faces in each race by Face++.

Race	Faces in female only images	Faces in male only images	Face in images with both genders	Total faces
**Black**	33 (49.3%)	17 (25.4%)	17 (25.4%)	67
**White**	289 (42.4%)	97 (14.2%)	296 (43.4%)	682
**Asian**	38 (44.2%)	17 (19.8%)	31 (40.8%)	86
**Ambiguous**	81 (49.4%)	24 (14.6%)	59 (36.0%)	164

Table 4: The demographic distribution for youth and adult by Face++.

Gender	Age	Black	White	Asian	Ambiguous
**Female**	Youth	15 (6.4%)	140 (59.3%)	27 (11.4%)	54 (22.9%)
	Adult	18 (9.7%)	135 (73.0%)	11 (5.9%)	21 (11.4%)
**Male**	Youth	5 (9.8%)	30 (58.8%)	12 (23.5%)	4 (7.8%)
	Adult	12 (12.5%)	62 (64.6%)	5 (5.2%)	17 (17.7%)
**Both genders**	Youth	14 (5.8%)	174 (71.9%)	21 (8.7%)	33 (13.6%)
	Adult	3 (2.7%)	88 (77.9%)	4 (3.5%)	18 (15.9%)

Table 5: The gender, age, and race distribution in different user group by Face++.

		Government-health user					Organization-health user					Organization-news user					Individuals (any topic)				
**Gender**	**Age**	**T(#)**	**B**	**W**	**A**	**AM**	**T(#)**	**B**	**W**	**A**	**AM**	**T(#)**	**B**	**W**	**A**	**AM**	**T(#)**	**B**	**W**	**A**	**AM**
**Female**	**youth**	56	5%	66%	7%	21%	26	15%	58%	4%	23%	26	0%	46%	35%	19%	56	2%	68%	13%	18%
	**adult**	18	28%	56%	11%	6%	27	11%	74%	4%	11%	21	14%	57%	0%	29%	31	7%	77%	7%	10%
**Male**	**youth**	10	0%	30%	70%	0%	11	27%	64%	9%	0%	4	0%	50%	25%	25%	23	9%	65%	13%	13%
	**adult**	3	0%	67%	0%	33%	19	0%	68%	16%	16%	8	0%	88%	0%	13%	47	26%	66%	4%	4%
**Both genders**	**youth**	37	5%	62%	3%	30%	60	2%	70%	8%	20%	4	0%	50%	0%	50%	45	2%	78%	11%	9%
	**adult**	22	0%	86%	5%	9%	26	4%	77%	4%	15%	5	0%	80%	0%	20%	36	6%	75%	3%	17%

T: Total.
B: black.
W: white.
A: Asian.
AM: Ambiguous.

References:

1. Census data. Retreived from: https://www.census.gov/quickfacts/fact/table/US/PST045217
2. Census data. Retreived from: https://www.census.gov/quickfacts/fact/table/US/PST045216
3. HPV-Associated Cancers Rates by Race and Ethnicity. Retrieved from: https://www.cdc.gov/cancer/hpv/statistics/race.htm
