# Supplementary material for: Discordance Between Human Papillomavirus Twitter Images and Disparities in Human Papillomavirus Risk and Disease in the United States: Mixed-Methods Analysis
Source: J Med Internet Res. 2018 Sep 14;20(9):e10244. doi: 10.2196/10244 (PMC6231890; doi:10.2196/10244)
Supplement: Multimedia Appendix 2 [file jmir_v20i9e10244_app2.pdf]

Discordance between HPV Twitter Images and Disparities in HPV Risk and Disease: Mixed Methods Analysis

Multimedia Appendix 2

Table 1: The gender, age, and race distribution in different source group by manual annotation.

		Government-health user					Organization-health user					Organization-news user					Individuals (any topic)				
**Gender**	**Age**	**T(#)**	**B**	**W**	**A**	**AM**	**T(#)**	**B**	**W**	**A**	**AM**	**T(#)**	**B**	**W**	**A**	**AM**	**T(#)**	**B**	**W**	**A**	**AM**
**Female**	**youth**	24	25%	42%	13%	21 %	38	18%	63%	3%	16%	29	3%	97%	0%	0%	55	6%	80%	2%	13%
	**adult**	20	20%	55%	15%	10 %	49	14%	61%	6%	18%	37	5%	78%	5%	11%	62	21%	63%	7%	10%
**Male**	**youth**	15	0%	80%	0%	20 %	3	100 %	0%	0%	0%	1	0%	0%	0%	100%	7	29%	71%	0%	0%
	**adult**	1	0%	100%	0%	0%	14	0%	79%	14%	7%	12	0%	100%	0%	0%	30	3%	47%	0%	50%
**Both genders**	**youth**	92	12%	55%	2%	30 %	96	8%	71%	6%	15%	10	10%	60%	0%	30%	50	10%	64%	2%	24%
	**adult**	62	5%	89%	0%	7%	30	3%	83%	7%	7%	9	22%	78%	0%	0%	52	2%	54%	15%	29%

T: Total.
B: Black.
W: White.
A: Asian.
AM: Ambiguous.
